# Concordance of Actionable Driver Alterations Between Primary Lung Adenocarcinoma and Paired Thoracic Metastases: A Prospective Next-Generation Sequencing Study

**DOI:** 10.3390/cancers18111773

**Published:** 2026-05-28

**Authors:** Luca Bertolaccini, Mariano Lombardi, Matteo Chiari, Alessandra Rappa, Monica Casiraghi, Marianna D’Ercole, Antonio Mazzella, Giorgio Lo Iacono, Shehab Mohamed, Valeria Midolo De Luca, Nicola Fusco, Elena Guerini Rocco, Lorenzo Spaggiari

**Affiliations:** 1 Department of Oncology and Hemato-Oncology, University of Milan, Via Santa Sofia 9/1, 20122 Milan, Italy; 2 Department of Thoracic Surgery, IEO, European Institute of Oncology IRCCS, Via Ripamonti 435, 20141 Milan, Italy; 3 Division of Pathology, IEO, European Institute of Oncology IRCCS, Via Ripamonti 435, 20141 Milan, Italy

**Keywords:** lung cancer, pleural metastasis, molecular concordance, next-generation sequencing, tumor heterogeneity, paired biopsy

## Abstract

Background: The molecular concordance between primary lung adenocarcinoma and metastatic lesions remains incompletely characterized despite its direct implications for precision oncology and biopsy-driven therapeutic decision-making. This prospective monocentric paired-sample study evaluated genomic concordance between primary lung adenocarcinoma and synchronous thoracic metastatic lesions using targeted next-generation sequencing (NGS). Methods: We identified 27 treatment-naïve patients with histologically confirmed lung adenocarcinoma who underwent paired molecular profiling of the primary tumor and a synchronous thoracic metastatic site (pleural or intrapulmonary). DNA and RNA were analyzed using validated institutional NGS platforms. Genomic alterations, including clinically actionable oncogenic drivers consistently covered by the sequencing panel used in each pair, were compared across matched samples. Concordance was assessed using exact binomial confidence intervals, Cohen’s κ statistics, McNemar tests, and paired Wilcoxon signed-rank tests. Results: Actionable driver alterations were identified in 17 of 27 patients (63.0%; 95% CI 42.4–80.6), including *EGFR* mutations (40.7%), *KRAS* alterations (18.5%), and one *ALK* gene rearrangement (3.7%). *TP53* concurrent mutations were detected in 14 cases (51.9%). Across all 27 paired samples, driver-level concordance was 100% (95% CI 87.2–100), with perfect agreement for *EGFR*, *KRAS*, and *ALK* alterations (κ = 1.00). *TP53* mutations showed high concordance (92.9%; κ = 0.85), while CNVs were concordant in 88.0% of evaluable pairs. Variant allele frequency (VAF) comparisons, adjusted for tumor cellularity, further supported the apparent clonal stability of driver alterations across paired samples. Conclusions: This study demonstrates very high molecular concordance between primary lung adenocarcinomas and their synchronous pleural or intrapulmonary metastases. The observed 100% concordance of actionable driver alterations across paired specimens supports the clinical reliability of thoracic metastatic biopsies for baseline molecular profiling in treatment-naïve disease. Although limited by sample size, these findings support the biological stability of actionable driver alterations during early thoracic metastatic dissemination.

## Simple Summary

Lung adenocarcinoma is frequently diagnosed at an advanced stage, where molecular profiling plays a crucial role in guiding targeted therapies. In many patients, metastatic thoracic lesions such as pleural or intrapulmonary metastases represent the only accessible tissue for genomic testing. However, the reliability of metastatic samples compared with the primary tumor remains incompletely defined. In this prospective paired-sample study, we analyzed matched primary lung adenocarcinomas and synchronous thoracic metastatic lesions using next-generation sequencing. We observed a very high concordance of actionable genomic alterations, including EGFR, KRAS, and ALK driver events, between primary and metastatic sites. These findings suggest that thoracic metastatic biopsies can provide clinically reliable molecular information for treatment selection in treatment-naïve patients with lung adenocarcinoma. Although the cohort size was limited, the results support the biological stability of major oncogenic drivers during early thoracic metastatic dissemination and reinforce the clinical utility of metastatic tissue sampling in precision oncology.

## 1. Introduction

Pleural and pulmonary metastases from lung cancer represent a biologically heterogeneous condition whose molecular determinants remain only partially elucidated. Although significant advances in genomic characterization of non-small cell lung cancer (NSCLC) have transformed the therapeutic landscape, most available evidence derives from primary tumors. In contrast, the molecular profile of metastatic lesions, particularly pleural metastases, has been far less systematically explored [1].

Several studies have demonstrated that metastatic deposits may acquire additional driver alterations not present in the primary tumor, reflecting spatial and temporal genomic heterogeneity and potentially influencing therapeutic decision-making and resistance mechanisms [2,3,4]. This phenomenon has been described across multiple driver genes, including Epidermal Growth Factor Receptor (EGFR), Kirsten Rat Sarcoma Viral Oncogene Homolog (KRAS), Anaplastic Lymphoma Kinase (ALK), v-Raf Murine Sarcoma Viral Oncogene Homolog B1 (BRAF), and Mesenchymal–Epithelial Transition Factor (MET), with discordance rates ranging from 10% to 30% across molecular pathways [5,6,7,8]. From a clinical perspective, pleural or intrapulmonary metastatic lesions are frequently the only accessible tissue in advanced disease. Consequently, therapeutic decisions may rely exclusively on metastatic biopsies rather than on the primary tumor. Demonstrating genomic concordance between these sites is therefore essential to validate the clinical reliability of metastatic sampling for precision oncology [9,10,11]. In advanced lung adenocarcinoma, thoracic metastatic lesions frequently represent the only safely accessible tissue source for molecular testing. Consequently, therapeutic decisions may rely exclusively on metastatic biopsies rather than on primary tumor specimens. In this context, the clinical relevance of concordance analysis lies not in the biological novelty of canonical oncogenic drivers themselves, but in validating whether metastatic-site molecular profiling can reliably guide precision oncology strategies. However, prospective paired-sample analyses specifically focusing on synchronous thoracic metastatic sites remain limited, particularly among treatment-naïve patients undergoing clinically indicated thoracic procedures.

Thoracic metastatic lesions represent a clinically relevant and biologically informative compartment in lung cancer evolution, as pleural or intrapulmonary deposits are frequently sampled during diagnostic thoracoscopy or Video-Assisted Thoracic Surgery (VATS) procedures and may represent the only available tissue for molecular profiling. Spatial heterogeneity in lung cancer reflects the branching evolutionary architecture of tumors, in which early truncal driver events are preserved across metastatic lineages. In contrast, later subclonal alterations may diverge between tumor sites [12].

This monocentric prospective pilot paired-sample study aimed to evaluate whether synchronous thoracic metastatic lesions can reliably reproduce the actionable molecular profile of primary lung adenocarcinoma and therefore serve as clinically reliable surrogate specimens for baseline molecular testing [13]. Unlike many previous retrospective studies, which included heterogeneous metastatic sites and used older molecular techniques, the present study specifically focused on prospectively collected synchronous thoracic metastatic lesions and analyzed them using contemporary, clinically validated next-generation sequencing platforms in treatment-naïve patients [11,14,15,16,17]. By integrating clinical, pathological, and short-term outcome data with paired molecular profiles, this work seeks to provide a clearer picture of the biological trajectories that shape metastatic spread in NSCLC.

## 2. Materials and Methods

### 2.1. Study Population

Patients with radiologically suspected or histologically confirmed lung cancer and evidence of pleural or parenchymal metastases, defined radiologically and/or pathologically according to International Association for the Study of Lung Cancer (IASLC) criteria for intrathoracic metastatic disease, were prospectively screened for eligibility [18]. Patients were prospectively enrolled between June 2023 and December 2025. Patients were included if they were aged 18 years or older, had either a resected primary tumor or a primary lesion suitable for sampling, had at least one metastatic intrathoracic site accessible for biopsy, and had sufficient tissue available for molecular profiling. Patients were excluded if no paired tissue could be obtained within a maximum interval of 4 weeks, if there was no intervening systemic therapy, or if tissue quality was inadequate for sequencing. The principal reasons for exclusion included insufficient tissue quality for sequencing, inadequate tumor cellularity, absence of synchronous paired samples, or incomplete molecular profiling.

All procedures were performed in accordance with institutional standards. Paired tumor samples were obtained from each patient whenever feasible. Sampling of primary and metastatic lesions occurred during the same diagnostic procedure or within a clinically negligible time interval, ensuring that paired specimens reflected synchronous disease biology rather than temporal tumor evolution. Primary tumors and metastatic lesions were sampled through surgical resection or VATS biopsy. Dedicated thoracic pathologists reviewed all specimens to confirm the diagnosis of adenocarcinoma, tumor cellularity, and adequacy for molecular testing.

Clinical variables were collected, including demographics, smoking status, Eastern Cooperative Oncology Group (ECOG) performance status [19], TNM stage [20], prior oncologic treatments, timing and site of metastasis, and procedural characteristics. Short-term outcomes such as postoperative complications, length of stay, and 30-day and 90-day mortalities were recorded. Follow-up was performed according to routine institutional protocols, and exploratory survival data were captured when available.

All patients provided written informed consent, and the study was conducted in accordance with the Declaration of Helsinki and institutional ethical guidelines. Our Institutional Research Board approved the study (IEO 1956, 5 June 2023).

### 2.2. Molecular Analysis

Molecular analysis was performed using next-generation sequencing (NGS) panels to detect mutations, copy-number variants (CNVs), and fusion genes. TruSight Oncology 500 (Illumina, San Diego, CA, USA), Oncomine Dx Express (Thermo Fisher Scientific, Waltham, MA, USA), and Oncomine Comprehensive Assay (Thermo Fisher Scientific, Waltham, MA, USA) were used in accordance with institutional clinical diagnostic workflows at the time of testing. Although multiple clinically validated panels were used over the study period, all assays included the core actionable NSCLC driver genes recommended by international molecular testing guidelines, ensuring comparability of driver-level analyses across patients. The selection of actionable driver genes was based on contemporary international molecular testing recommendations for advanced NSCLC [21]. For each patient, the primary tumor and matched metastatic lesion were analyzed using the same sequencing platform and bioinformatics pipeline to minimize technical bias in the concordance assessment. This strategy was specifically adopted to minimize intra-pair technical variability and reduce potential platform-related analytical bias. All panels covered the main actionable lung cancer drivers [EGFR, KRAS, ALK, ROS Proto-Oncogene 1, Receptor Tyrosine Kinase (ROS1), BRAF, MET, Erb-B2 Receptor Tyrosine Kinase 2 (ERBB2), Neuregulin 1 (NRG1), Neurotrophic Tyrosine Receptor Kinase 1, 2, and 3 (NTRK1/2/3), and Rearranged during Transfection (RET)], ensuring comparability of driver-level analyses across patients. However, not all alterations were identified in the present cohort. Driver-level concordance analyses were restricted to genes and variant classes consistently captured by the panel for each pair (single-nucleotide variants [SNVs]/insertions–deletions [indels], copy-number variations [CNVs], and gene fusions as applicable). Variant calling was conducted using validated bioinformatic pipelines, using minimum depth and allele frequency thresholds consistent with clinical sequencing standards (≥500× read depth and variant allele frequency ≥ 5%). Variant calling required a minimum sequencing depth of 500× and a minimum variant allele frequency threshold of 5%. Quality-control filtering and annotation were performed using institutionally validated bioinformatics pipelines in accordance with routine molecular diagnostic workflows. Because variant allele frequency (VAF) is influenced by tumor purity and local copy-number state, comparisons of VAF between paired primary and metastatic samples were normalized according to tumor cellularity. Normalized VAF values were calculated as: Normalized VAF = Observed VAF/Tumor cellularity. Tissue tumor cellularity was independently estimated by dedicated thoracic pathologists through routine histopathological assessment of hematoxylin–eosin-stained slides before sequencing. This normalization was intended solely as an approximate descriptive adjustment and not as a formal purity-corrected clonality estimate.

### 2.3. Statistical Analysis

Concordance was defined as the presence of the same actionable driver alteration (gene and variant class) in both the primary tumor and the matched metastatic specimen. It was assessed at the patient and gene levels, considering only clinically actionable driver alterations included in the sequencing panels. In contrast, discordance was recorded when at least one clinically relevant alteration was present exclusively in one of the paired specimens. Driver-level concordance was evaluated at the patient level, meaning that concordance required the preservation of the dominant oncogenic driver between paired samples, regardless of secondary alterations.

The primary endpoint was the proportion of patients demonstrating molecular concordance between primary tumors and matched metastatic lesions. This proportion was expressed with exact 95% confidence intervals for the binomial distribution. Agreement between paired samples regarding individual gene alterations was assessed using Cohen’s kappa coefficients and interpreted according to established qualitative thresholds. Agreement statistics were interpreted descriptively due to the exploratory nature of the study and the relatively small cohort size. For paired categorical comparisons of mutational status between primary and metastatic sites, McNemar’s test was planned to evaluate discordance patterns. However, because no discordant actionable driver alterations were observed, formal McNemar testing was not informative for driver-level comparisons, and concordance results are therefore reported descriptively. Associations between concordance or discordance and clinical–pathological variables were examined using chi-squared or Fisher’s exact tests for categorical variables and Student’s *t* test or Mann–Whitney U test for continuous variables, depending on distribution. Given the exploratory design and limited sample size, all inferential analyses should be interpreted as descriptive and hypothesis-generating.

No formal power calculation was performed. This reflects the exploratory pilot nature of the study and the rarity of prospectively collected paired thoracic metastatic samples suitable for molecular analysis. The planned sample size of 25 patients reflected the study’s exploratory nature. It aligned with similar paired-sample genomic studies in lung cancer, which typically involve small cohorts due to the procedural invasiveness and the rarity of obtaining paired tissues. Given the exploratory design and limited cohort size, statistical analyses should be interpreted primarily as descriptive measures of concordance rather than as hypothesis-testing inferential statistics.

All statistical analyses were performed using R (Version 4.5.1, R Foundation for Statistical Computing, Vienna, Austria). All tests were two-sided, and *p*-values are reported for descriptive purposes [22,23].

## 3. Results

A total of 27 patients with histologically confirmed lung adenocarcinoma were included in the study. The median age was 62 years (IQR 53–70), and 15 patients (56%) were female. Paired primary and metastatic samples were available for all patients. Metastatic sites included pleural deposits in 17 cases (63.0%) and intrapulmonary metastases in 10 cases (37.0%), identified either during diagnostic thoracoscopy or during surgical procedures. Tumor cellularity ranged from <20% to approximately 60%, with lower purity more frequently observed in pleural samples. Sequencing platforms included Oncomine Comprehensive Assay v3 in 22 patients, Oncomine Dx Express in four patients, and TruSight Oncology 500 in one patient. The baseline clinical and pathological characteristics of the study population are summarized in Table 1.

Given the limited sample size, exploratory subgroup analyses by demographic or clinicopathological variables were interpreted descriptively and did not reveal statistically meaningful differences in concordance patterns. Actionable oncogenic drivers were identified in 17 of 27 patients (63.0%; 95% CI 42.4–80.6). EGFR alterations were the most frequent driver events, detected in 11 patients (40.7%), including exon 19 deletions and exon 21 L858R substitutions. KRAS mutations were present in five patients (18.5%), most commonly affecting codon 12. One patient (3.7%) harbored an EML4–ALK fusion transcript. No clinically actionable alterations were identified in BRAF, MET exon 14 skipping, ROS1, RET, ERBB2, or NTRK genes. The distribution of actionable driver alterations detected in primary tumors is summarized in Table 2. TP53 alterations were reported separately as recurrent co-mutations rather than canonical actionable driver alterations.

Tumor suppressor alterations were common. TP53 mutations were detected in 14 patients (51.9%), with hotspot residues such as p.Arg175His and splice-site variants most common. Additional genomic alterations included copy-number gains in genes such as CCNE1, AKT2, CDK6, RICTOR, and MDM2.

Paired comparison between primary tumors and thoracic metastases demonstrated high genomic concordance across the analyzed driver alterations. All actionable driver alterations detected in primary tumors were preserved in the matched metastatic specimens, resulting in a driver-level concordance of 100% (27/27; 95% CI 87.2–100) across all evaluable pairs. Agreement for driver mutations was therefore perfect (Cohen’s κ = 1.00). TP53 mutations also showed high concordance between paired samples (92.9%; κ = 0.85). Copy-number alterations were concordant in 88% of evaluable cases. Detailed concordance metrics for each genomic category are reported in Table 3.

**Figure 1:**
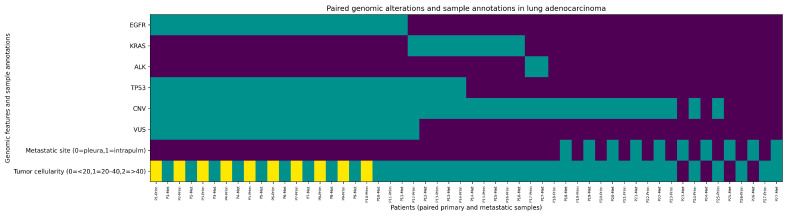
Heatmap of genomic alterations in paired primary lung adenocarcinomas and matched thoracic metastatic lesions. For each patient, the primary tumor and matched metastatic lesion are displayed as adjacent columns to facilitate direct visual comparison of genomic alterations. The figure was generated at high resolution to improve the readability of individual sample annotations. Rows represent actionable driver alterations and recurrent secondary genomic events. Alterations are grouped according to genomic category (driver mutations, tumor suppressor mutations, and copy-number alterations). Color intensity reflects normalized variant allele frequency levels, with darker shades corresponding to higher relative allele frequencies.

Variant allele frequency analysis supported the clonal stability of driver mutations (Figure 1). A linear regression analysis of normalized variant allele frequencies between primary and metastatic lesions demonstrated a strong quantitative correlation across paired samples. Median VAF values were lower in metastatic lesions than in primary tumors (approximately 26% versus 47%), largely reflecting differences in tumor cellularity rather than biological divergence. After normalization for tumor cellularity, VAF values remained broadly consistent across paired samples, supporting the interpretation that actionable driver alterations represent stable clonal events during early metastatic dissemination. A detailed comparison of observed and tumor-cellularity-normalized variant allele frequencies between paired samples is provided in Table 4. In all patients harboring actionable genomic alterations, molecular profiling results were discussed in the institutional multidisciplinary thoracic oncology tumor board and directly informed treatment selection in accordance with contemporary precision oncology guidelines.

## 4. Discussion

In this prospective pilot cohort of 27 patients with lung adenocarcinoma, we observed very high molecular concordance between primary tumors and paired thoracic metastatic lesions, with preservation of all actionable oncogenic drivers across paired samples. These findings suggest that the actionable genomic architecture remains remarkably stable in synchronous thoracic metastases, supporting the assumption that a single-site biopsy, whether of the primary tumor or metastatic focus, provides clinically relevant molecular information for targeted therapeutic decision-making. The importance of this observation is primarily clinical rather than biologically unexpected. Although dominant actionable drivers such as EGFR, KRAS, and ALK are widely considered early truncal oncogenic events, thoracic metastatic lesions are frequently the only available tissue source for molecular diagnostics in advanced disease. Demonstrating concordance, therefore, directly supports the reliability of metastatic-site molecular profiling for therapeutic decision-making.

Our observations align with and extend previous studies evaluating spatial genomic heterogeneity in NSCLC. Early multi-region sequencing analyses, such as those from the TRACERx program [3,24], demonstrated that although substantial intratumoral heterogeneity exists, clonal mutations (particularly *EGFR*, *KRAS*, *ALK* fusions, and key tumor suppressor alterations) are typically shared across tumor regions [4,25]. More than 75% of actionable drivers in NSCLC are early clonal events that persist across metastatic evolution [25]. Our data support a similar model of evolutionary constraint, in which, despite variable tumor cellularity and diverse sampling sites, driver-level concordance was observed.

Studies specifically examining paired primary–metastatic samples have reported variable discordance rates, often due to methodological limitations, small sample sizes, or heterogeneous metastatic sites. In 15 paired NSCLC samples, a 13% discordance rate for *EGFR* and *KRAS* mutations was observed [26]. Others reported discordance rates of 10–28% across genes [17]. However, many discrepancies in earlier studies were due to technical challenges, such as the exclusive use of PCR and low-yield biopsies, or lower-sensitivity sequencing platforms. Nevertheless, prior studies have still reported variable discordance rates attributable to tumor heterogeneity, clonal evolution, sampling variability, and technical limitations, supporting the continued clinical relevance of prospective concordance analyses. Our validated NGS workflows and matched-panel sequencing likely contributed to the absence of false-negative calls, particularly in low-cellularity pleural metastases. The absence of discordant driver alterations supports the notion that actionable oncogenic drivers in lung adenocarcinoma typically represent early truncal events that are maintained during early metastatic dissemination.

Pleural lesions may reflect advanced subclonal diversification [9,10]. Consistent with our findings, studies of malignant pleural effusion have reported high detection rates of canonical driver mutations, particularly *EGFR* alterations, supporting the idea that pleural disease reliably reflects the genomic profile of the primary tumor.

Analyses performed in European cohorts have demonstrated that EGFR mutations are frequently detectable in malignant pleural effusions from lung adenocarcinoma and may even be enriched relative to primary tumor samples, likely reflecting tumor-cell shedding into the pleural space [27]. Similarly, molecular characterization of pleural effusion specimens has consistently reported high rates of EGFR mutations, with strong concordance with the primary tumor genotype, supporting the reliability of pleural-derived material for molecular diagnostics [28]. In our cohort, EGFR alterations represented the most prevalent actionable driver and were particularly frequent among patients with pleural involvement. This pattern aligns with previous observations suggesting that EGFR-driven tumors may have a biological propensity for pleural dissemination. In contrast, KRAS mutations were less frequently observed in pleural metastatic samples. These findings reinforce the concept that EGFR-mutant lung adenocarcinomas commonly present with pleural disease and that pleural specimens can reliably capture the dominant oncogenic drivers of the tumor [27].

EGFR-mutated cases in our series also demonstrated remarkable molecular stability between primary and metastatic sites, consistent with previous reports showing >95% concordance in EGFR-mutant NSCLC across distinct metastatic deposits [17]. EGFR exon 19 deletions and L858R mutations, both dominant truncal drivers in lung adenocarcinoma, were retained across paired samples. The observed differences in variant allele frequencies likely reflect variations in tumor cellularity rather than true biological divergence, supporting the absence of selective sweeps or subclonal outgrowth during pleural dissemination. Although the present study was not specifically designed for comprehensive phylogenetic reconstruction or single-cell evolutionary analysis, the observed preservation of actionable driver alterations supports current models suggesting that dominant oncogenic drivers frequently represent early truncal events maintained during metastatic dissemination.

*TP53* mutations and copy-number alterations exhibited slightly lower concordance than actionable drivers. This lower concordance likely reflects both technical and biological factors. From a technical perspective, CNV detection is highly sensitive to tumor cellularity, stromal contamination, and sequencing depth, particularly in low-purity pleural specimens. From a biological perspective, CNVs may represent later, more heterogeneous subclonal evolutionary events than early truncal driver mutations such as EGFR or KRAS alterations. However, the overall agreement remained high (92.9% for TP53; 88% for CNVs). These rates mirror findings from metastatic sequencing studies, in which copy-number variability often reflects technical variation or differences in tumor cellularity, namely, the proportion of tumor cells within the analyzed specimen [29]. No statistically meaningful differences in VAF distributions were observed after accounting for tumor cellularity. The fact that the rare discordant findings were confined to samples with reduced tumor cellularity further supports the interpretation that most actionable driver alterations represent early truncal events. This observation further supports the possibility that at least part of the observed CNV discordance may derive from reduced analytical sensitivity rather than from true biological divergence. In contrast, apparent discrepancies may arise from technical limitations related to specimen adequacy.

The genomic stability demonstrated in this work has direct clinical consequences. In the present cohort, actionable molecular findings directly informed multidisciplinary therapeutic decision-making, supporting the practical clinical utility of metastatic-site molecular profiling. Either primary or metastatic tissue is suitable for molecular diagnostics, which is relevant when pleural biopsies are performed for diagnostic palliation or when the primary tumor is inaccessible. Since actionable drivers are fully preserved across samples, treatment decisions based on a single lesion can be made with high confidence. Pleural or intrapulmonary biopsies obtained during diagnostic thoracoscopy or VATS procedures can therefore be considered adequate for baseline genomic testing in treatment-naïve lung adenocarcinoma. Taken together, our findings support the concept that actionable driver alterations in lung adenocarcinoma represent early truncal genomic events that are preserved during early metastatic dissemination. This observation reinforces the biological robustness of current precision oncology strategies in which therapeutic decisions are based on the identification of dominant oncogenic drivers rather than on extensive spatial sampling.

### Limitations

This study has several limitations. Most importantly, the relatively small sample size requires caution in interpreting the observed complete driver-level concordance, as confidence intervals remain wide despite the absence of discordant actionable alterations. The sample size was modest, though comparable to or larger than that of most published paired primary–metastatic sequencing studies in lung cancer [30]. The systematic acquisition of paired tissue partially mitigates this issue, yet the confidence intervals around some concordance estimates remain wide. This monocentric design and the requirement for paired tissue availability may introduce selection bias toward patients undergoing invasive diagnostic procedures. Tumor cellularity estimates were based on pathological assessment rather than on computational inference of tumor purity. Second, while high-depth NGS was applied uniformly, four metastatic samples exhibited suboptimal tumor cellularity (<20%), which may reduce sensitivity for low-frequency subclonal variants. In this context, the few observed discrepancies in secondary alterations were observed in specimens with lower tumor cellularity, reinforcing the possibility that reduced tumor purity may limit variant detection rather than reflect genuine genomic divergence between primary and metastatic lesions. The preservation of all actionable drivers in these cases suggests that the principal conclusions are robust, but subtle evolutionary events below the limit of detection cannot be excluded. Third, this cohort consisted exclusively of treatment-naïve patients. Consequently, the study cannot address molecular evolution under therapeutic pressure, particularly the emergence of resistance mechanisms. The stability observed here, therefore, reflects baseline disease biology rather than the dynamics of post-therapy adaptation. Fourth, the study focused on pleural and intrapulmonary metastatic sites; extrapolation to distant organ metastases (e.g., brain, liver, bone) should be approached with caution, as prior studies have suggested greater heterogeneity in specific non-thoracic sites. The use of targeted NGS panels may underestimate genomic heterogeneity outside predefined cancer genes. Whole-exome or single-cell sequencing could identify additional subclonal alterations not captured by targeted panels. Publicly available datasets containing prospectively collected paired primary lung adenocarcinoma and synchronous thoracic metastatic lesion sequencing data remain limited, precluding reliable external validation analyses [31]. Despite these limitations, the study provides strong prospective evidence for molecular profile concordance between primary and thoracic metastatic lesions in lung adenocarcinoma, reinforcing the reliability of single-site biopsy for clinical decision-making. Future multicenter collaborative studies with larger cohorts will be necessary to validate these findings externally.

## 5. Conclusions

This prospective pilot paired-sample analysis demonstrated very high concordance of actionable driver alterations between primary lung adenocarcinomas and synchronous thoracic metastatic lesions, supporting the potential clinical reliability of thoracic metastatic biopsies for baseline molecular profiling in treatment-naïve disease. These data suggest that, in treatment-naïve disease, synchronous thoracic metastatic lesions may reliably reproduce the dominant actionable molecular profile of the primary tumor and may therefore represent clinically informative surrogate specimens for baseline molecular profiling. Tumor suppressor mutations and copy-number profiles likewise demonstrated high agreement. Nevertheless, given the relatively limited cohort size and monocentric design, these findings should be interpreted as exploratory and hypothesis-generating pending external multicenter validation.

## Figures and Tables

**Table 1:** Baseline Characteristics of the Study Population.

Variable	Value
**Age, median (IQR)**	62 (52–69)
**Sex, n (%)**	Female 15 (56%) Male 12 (44%)
**Smoking status, n (%)**	Former smoker 15 (57%) Never smoker 7 (26%) Current smoker 5 (19%)
**Histology**	Adenocarcinoma (100%)
**Disease status**	Synchronous metastatic
**Metastatic site sampled, n (%)**	Pleura 17 (63%) Intrapulmonary metastasis 10 (37%)
**Tumor cellularity**	<20% (n = 4) 20–60% (n = 23)
**Sequencing platform**	Oncomine OCA v3 (n = 22) Oncomine Dx Express (n = 4) TruSight Oncology 500 (n = 1)

**Table 2:** Actionable Driver Alterations in Primary Tumors.

Gene/Alteration	No. (%)
**EGFR**	11 (40.7%)
**KRAS**	5 (18.5%)
**ALK (EML4–ALK fusion)**	1 (3.7%)
**BRAF V600**	0
**MET exon 14 skipping**	0
**ROS1/RET/NTRK1–3**	0
**TP53 co-mutation**	14 (51.9%)

**Table 3:** Concordance Between Primary and Metastatic Samples. * McNemar test.

Molecular Feature	Concordance (%)	95% CI	Cohen’s Kappa	*p*-Value
**Actionable drivers (EGFR/KRAS/ALK)**	100% (27/27)	87.2–100	1.00	–
**TP53**	92.9% (13/14)	66.1–99.8	0.85	–
**Copy-number alterations**	88% (22/25)	68.8–97.5	0.78	–
**Variants of uncertain significance**	91.7% (11/12)	61.5–99.8	–	0.34 *

**Table 4:** Observed and tumor-cellularity-normalized Variant Allele Frequency (VAF) comparison between primary tumors and matched thoracic metastatic lesions. Normalized VAF values were approximated by dividing the observed VAF by tumor cellularity. Values exceeding 1.0 may occur in samples with copy-number gain or uncertainty in tumor purity estimation. They should therefore be interpreted as approximate indicators of clonal representation rather than exact allele fractions.

Mutation	Primary VAF (%)	Tumor Cellularity Primary	Normalized VAF Primary	Metastatic VAF (%)	Tumor Cellularity Metastasis	Normalized VAF Metastasis	Δ Normalized
**EGFR Ex19del**	42	0.50	0.84	22	0.20	1.10	+0.26
**EGFR L858R**	52	0.60	0.87	28	0.30	0.93	+0.06
**KRAS G12V**	5	0.50	0.10	5	0.50	0.10	0
**KRAS G12D**	19	0.60	0.32	14	0.60	0.23	−0.09
**TP53 R175H**	44	0.50	0.88	11	0.20	0.55	−0.33
**TP53 splice**	54	0.60	0.90	23	0.30	0.77	−0.13
**ALK fusion**	Qualitative	—	—	Qualitative	—	—	—

## Data Availability

The raw data supporting the conclusions of this article will be made available by the authors on request.
